# Carotid Intima-Media Thickness Progression in HIV-Infected Adults Occurs Preferentially at the Carotid Bifurcation and Is Predicted by Inflammation

**DOI:** 10.1161/JAHA.111.000422

**Published:** 2012-04-24

**Authors:** Priscilla Y. Hsue, Rebecca Scherzer, Peter W. Hunt, Amanda Schnell, Ann F. Bolger, S.C. Kalapus, Kristinalisa Maka, Jeffrey N. Martin, Peter Ganz, Steven G. Deeks

**Affiliations:** From the Divisions of Cardiology, University of CaliforniaSan Francisco (P.Y.H., A.S., A.F.B., K.M., P.G., S.C.K.); Positive Health Program of the Department of Medicine, San Francisco General Hospital, University of CaliforniaSan Francisco (P.Y.H., S.G.D., J.N.M.); San Francisco Veterans Affairs Medical Center and Department of Medicine UCSF, University of CaliforniaSan Francisco (R.S.); Department of Epidemiology and Biostatistics, University of CaliforniaSan Francisco (J.N.M.)

**Keywords:** AIDS, carotid arteries, inflammation, atherosclerosis

## Abstract

**Background:**

Shear stress gradients and inflammation have been causally associated with atherosclerosis development in carotid bifurcation regions. The mechanism underlying higher levels of carotid intima-media thickness observed among HIV-infected individuals remains unknown.

**Methods and Results:**

We measured carotid intima-media thickness progression and development of plaque in the common carotid, bifurcation region, and internal carotid artery in 300 HIV-infected persons and 47 controls. The median duration of follow-up was 2.4 years. When all segments were included, the rate of intima-media thickness progression was greater in HIV-infected subjects compared with controls after adjustment for traditional risk factors (0.055 vs. 0.024 mm/year, *P*=0.016). Rate of progression was also greater in the bifurcation region (0.067 vs. 0.025 mm/year, *P*=0.042) whereas differences were smaller in the common and internal regions. HIV-infected individuals had a greater incidence of plaque compared with controls in the internal (23% vs. 6.4%, *P*=0.0037) and bifurcation regions (34% vs. 17%, *P*=0.014). Among HIV-infected individuals, the rate of progression in the bifurcation region was more rapid compared with the common carotid, internal, or mean intima-media thickness; in contrast, progression rates among controls were similar at all sites. Baseline hsCRP was elevated in HIV-infected persons and was a predictor of progression in the bifurcation region.

**Conclusions:**

Atherosclerosis progresses preferentially in the carotid bifurcation region in HIV-infected individuals. hsCRP, a marker of inflammation, is elevated in HIV and is associated with progression in the bifurcation region. These data are consistent with a model in which the interplay between hemodynamic shear stresses and HIV-associated inflammation contribute to accelerated atherosclerosis. **(*J Am Heart Assoc*. 2012;1:jah3-e000422 doi: 10.1161/JAHA.111.000422.)**

**Clinical Trial Registration:**

URL: http://clinicaltrials.gov. Unique identifier: NCT01519141

## Introduction

Highly active antiretroviral therapy has allowed HIV-infected individuals to live longer and face a new set of health challenges. In particular, HIV-infected individuals have a higher prevalence of coronary artery disease, which has been demonstrated both using clinical end points^1–3^ and utilizing high-resolution ultrasound to assess carotid artery intima-media thickness (IMT).^4,5^ Although some studies have shown an increased carotid IMT in the setting of HIV infection,^4–6^ other studies have not confirmed this finding.^7,8^ These differences among studies may be due in part to limited sample size in some studies, different patient populations, and the method of imaging utilized by the investigators. The common carotid bifurcates into the internal and external carotid arteries; the bifurcation region is also referred to as the carotid bulb. Although most studies have focused on the more readily studied common carotid artery, fewer studies have also included assessment of the internal carotid and bifurcation regions.

Atherosclerotic lesions tend to occur at areas experiencing low endothelial shear stress which are local wall stresses that are generated by patterns of blood flow, such as the carotid bifurcation region.^9^ Low shear stress promotes atherosclerosis by a variety of mechanisms, including impairment of endothelial function by downregulation of eNOS and upregulation of endothelin-1,^10^ increased endothelial uptake of LDL,^11^ promotion of oxidative stress,^12,13^ and increased plaque thrombogenicity. Low endothelial shear stress also enhances the pro-atherogenic effect of chronic inflammation, because it allows for the attachment and infiltration of inflammatory cells via activation of NF-κB^14,15^ and subsequent upregulation of adhesion molecules, chemokines, and pro-inflammatory cytokines.^16^ Low endothelial shear stress has also been implicated in the transition of stable atherosclerotic lesions to vulnerable plaques resulting in acute coronary syndromes via a combination of vascular inflammation, change in the extracellular matrix, and wall remodeling.^17^

On the basis of emerging studies that untreated and treated HIV disease is associated with chronic inflammation^18–20^ and the observations that inflammation preferentially affects certain regions of the carotid,^9^ we hypothesized that HIV-infected individuals may exhibit more rapid IMT progression in the carotid bifurcation site compared with uninfected individuals. We therefore assessed carotid IMT in the common, internal, and bifurcation sites over time in a large cohort of HIV-infected and -uninfected adults. We also assessed carotid plaque and development of plaque during follow-up. We also hypothesized that chronic HIV-associated inflammation would be associated with accelerated atherosclerosis.

## Materials and Methods

### Participants

Study participants were obtained from the SCOPE cohort, which consists of patients followed at San Francisco General Hospital and the San Francisco Veteran's Affairs Medical Center. HIV infection status was confirmed using HIV antibody testing. Study participants were consecutive volunteers from the SCOPE study who agreed to participate in the study. Given our ongoing interest in defining the HIV-associated factors associated with cardiovascular risk independent of treatment toxicity and high-level viremia,^21^ we enriched our study population with rare individuals who controlled HIV replication indefinitely in absence of therapy (“elite” controllers).^22^ We categorized the cohort into four groups on the basis of their treatment status and virologic control at the baseline visit: (1) antiretroviral treated with undetectable viral loads using conventional assays (typically <40 to 50 copies/mL), (2) antiretroviral treated with detectable viremia, (3) antiretroviral untreated with detectable viremia (noncontrollers) and (4) antiretroviral untreated with undetectable viremia (elite controllers). Uninfected control subjects were recruited from San Francisco General Hospital, friends/acquaintances of HIV-infected study participants, and from advertisements placed around the hospital and San Francisco community. Controls were documented to be HIV-antibody negative before each IMT study. All participants were not preselected with respect to cardiovascular risk factors or coronary artery disease. The UCSF Committee on Human Research approved this study, and all individuals provided written informed consent before study enrollment.

### Clinical and Socio-Demographic Characteristics

Detailed interview and assessment of all study participants was performed by research staff at the time of enrollment. This assessment included ascertainment of cardiovascular disease (CVD) risk factors, past and present medication usage, and drug use. HIV disease characteristics including duration of HIV infection and current and prior antiretroviral medication were assessed in HIV-infected individuals.

### Laboratory Assays

Blood was drawn in the fasting state and used to measure total cholesterol, HDL cholesterol, and triglycerides. LDL-cholesterol was calculated by Friedewald's formula^23^ except for individuals who had triglycerides ≥4.56 mmol/L, where it was measured directly. C-reactive protein was measured using a high-sensitivity assay (Dade Behring, Deerfield, IL). The nadir CD4^+^ T cell count was defined as the lowest laboratory-confirmed value before the baseline IMT scan.

### Carotid Artery Intima-Media Thickness

We assessed the carotid IMT using the GE Vivid 7 system and a 10-MHz linear array probe as described previously.^5^ IMT was measured in 12 segments that included the near and far walls of the common carotid, bifurcation region, and internal carotid region on both the left and right sides, according to the standardized protocol of the Atherosclerosis Risk in Communities (ARIC) Study.^24,25^ The right and left carotid arteries were studied with the head in the midline position and tilted upward. The probe was then adjusted to obtain the near and far walls in a parallel orientation and then positioned to obtain the maximal luminal diameter in the longitudinal plane. Images were recorded digitally using a cine-loop format for subsequent analysis using manual caliper assessment of the digital images. Within each segment (common, internal, bifurcation region), IMT was calculated as the average of the near and far walls of the left and right carotid arteries. A single experienced technician who was blinded to the subjects’ HIV status performed all the IMT studies and caliper measurements of the digital images. Both external and internal landmarks were then identified and used at the subsequent visit to ensure that the measurements were performed at the same locations on the follow-up visits. Repeat scans and measurements were performed on 12 patients within 1 week and repeat measurements were performed on 15 patients; the mean absolute difference was 0.040 mm with a coefficient of variation of 3.4% and an intraclass correlation coefficient (ICC) of 0.98.

For all study participants, the median overall follow-up time (between baseline and the last available IMT measurement) was 2.4 years (interquartile range, IQR, 1.4–4.1, range: 0.6 to 7.2 years). Follow- time was variable as study enrollment is ongoing, and thus newly enrolled individuals have shorter follow-up. The median number of IMT procedures was 3 (IQR, 2–8); IMT studies were performed annually on both HIV-infected individuals and controls. The number of missing IMT segments at each site was lowest for common and highest for internal, and was slightly higher but not statistically different for HIV-infected compared with control participants (Table 1). Carotid plaque was defined as a focal region of IMT greater than 1.5 mm as recommended per the Society for Vascular Medicine (American Society of Echocardiography) guidelines.^26^

**Table 1.** Comparison of Missing IMT Segments at Each Site in HIV-Infected and HIV-Uninfected Participants

	Number of Segments	Baseline			Last Visit		
		
		HIV+	Control	*P*-value	HIV+	Control	*P*-value
Internal (4 total)	0 missing	233 (79%)	42 (89%)	0.34	261 (87%)	44 (94%)	0.17

	1 missing	38 (13%)	3 (6.4%)		18 (6.0%)	0	

	2 missing	19 (6.4%)	1 (2.1%)		19 (6.3%)	2 (4.3%)	

	3 missing	5 (1.7%)	1 (2.1%)		2 (0.7%)	1 (2.1%)	

	Total	295*	47		300	47	

Common (4 total)	0 missing	298 (99%)	47 (100%)	0.99	300 (100%)	47 (100%)	na

	1 missing	2 (0.7%)	0		0	0	

	2 missing	0	0		0	0	

	3 missing	0	0		0	0	

	Total	300	47		300	47	

Bulb (4 total)	0 missing	273 (91%)	46 (98%)	0.26	288 (96%)	45 (96%)	0.30

	1 missing	19 (6.3%)	0		11 (3.7%)	1 (2.1%)	

	2 missing	6 (2.0%)	1 (2.1%)		1 (0.3%)	1 (2.1%)	

	3 missing	2 (0.7%)	0		0	0	

	Total	300	47		300	47	

Mean (12 total)	0 missing	221 (74%)	42 (89%)	0.061	254 (85%)	43 (91%)	0.20

	1 missing	38 (13%)	3 (6.4%)		22 (7.3%)	0	

	2 missing	25 (8.3%)	0		15 (5.0%)	3 (6.4%)	

	≥3 missing	16 (5.3%)	2 (4.3%)		9 (3.0%)	1 (2.1%)	

	Total	300	47		300	47	

* Five HIV+ participants had no internal IMT segments at baseline. IMT, intima-media thickness: na, not applicable.

### Statistical Analysis

The following baseline characteristics were considered as candidates for inclusion in multivariable models: demographics, traditional CVD risk factors, HIV-related factors, comorbidities, and laboratory parameters. A limited number of characteristics were collected at follow-up visits, and were included as time-varying covariates in sensitivity analyses: HIV RNA, antiretroviral use, HIV disease category, and hsCRP. CVD risk factors included smoking (ever vs. never, duration, and current), diabetes, systolic and diastolic blood pressure, HDL and total cholesterol. HIV-related factors included HIV duration, HIV disease category, viral load, current and nadir CD4 count, prior opportunistic infection, and antiretroviral use. Comorbidities included hepatitis C, hypertension, antihypertensive medication, lipid lowering medication, testosterone medication, prior coronary artery disease/stroke, current aspirin usage, family history of CVD, and BMI. Laboratory parameters included hsCRP, triglycerides, and glucose. Right-skewed variables such as hsCRP and CD4 count were log-transformed to normalize their distributions. Smoothing splines were constructed using generalized additive models in order to examine the relationship of hsCRP with IMT.

The association of HIV status with carotid IMT levels and progression was assessed using linear mixed models, with random intercepts and slopes using a first-order antedependence covariance structure. Interaction terms between HIV status and time were used to determine whether the rate of progression in IMT differed between HIV-infected and -uninfected participants. The association of HIV status with prevalence of plaque at baseline was assessed using Poisson regression with a robust variance estimator, and the association of HIV status with risk of incident plaque was assessed using Cox proportional hazards models. We applied a staged modeling approach, first adjusting for demographic characteristics, next adjusting for traditional CVD risk factors (as listed above), and finally adjusting for hsCRP. “Interaction terms between each covariate (including HIV status) and time were used to determine and compare rates of change in IMT.”

Adjusted IMT progression was calculated for HIV-infected and -uninfected participants using population marginal means from a linear mixed model with dependent variable of IMT, containing all terms in the final model. To account for those without measured IMT at a given study visit due to loss to follow-up or drop-out, we performed sensitivity analyses using an inverse probability weighting approach by modeling each participant's probability of having measured IMT at each time point. Results were similar to the primary observed models (data not shown). All analyses were conducted using SAS version 9.2 (SAS Institute, Inc., Cary, NC).

## Results

### Baseline Characteristics

Carotid IMT was measured in 300 HIV-infected and 47 HIV-uninfected participants. On average, HIV-infected participants were older (median age 47 vs. 43), were more often African American, had a higher prevalence of smoking, hypertension, and dyslipidemia, and had higher levels of hsCRP (Table 2). The median duration of HIV infection was 13 years, and 70% of participants had used antiretroviral therapy for a median duration of 5 years. Baseline characteristics stratified by disease category are provided in Table 3.

**Table 2.** Baseline Characteristics of HIV-Infected and HIV−Uninfected Participants

ParameterHeadings	HIV-Infected (*n*=300)	HIV−uninfected (*n*=47)	*P*-value
Age (y)	47 (41–53)	43 (38–51)	0.048†

Gender			

Male	268 (89%)	38 (81%)	0.16

Female	29 (10%)	9 (19%)	

Transgender M->F	3 (1%)	0	

Race			

Caucasian	181 (60%)	36 (77%)	0.011†

African American	75 (25%)	3 (6%)	

Latino	30 (10%)	4 (9%)	

Other	14 (5%)	4 (9%)	

History of CAD/CVA	23 (8%)	1 (2%)	0.22

Cigarette smoking (ever)	207 (69%)	24 (51%)	0.020†

Diabetes mellitus	21 (7%)	1 (2%)	0.33

Hypertension	84 (28%)	4 (9%)	0.0035†

Hyperlipidemia treatment	51 (17%)	1 (2%)	0.0040†

LDL (mmol/L)	2.62 (2.08–3.23)	2.80 (2.21–3.90)	0.058

HDL (mmol/L)	1.05 (0.85–1.26)	1.26 (1.08–1.56)	<.0001†

TG (mmol/L)	1.64 (1.07–2.84)	0.99 (0.72–1.58)	<.0001†

T Chol (mmol/L)	4.69 (3.95–5.49)	4.77 (4.15–5.72)	0.34

hsCRP (nmol/L)	17.14 (7.62–41.91)	10.48 (3.81–29.52)	0.027†

BMI (kg/m^2^)	25 (23–28)	25 (23–30)	0.96

Hepatitis C virus antibody seropositivity	68 (23%)	0 (0%)	<0.0001†

Duration of HIV infection (y)	13 (8–17)		

ARV use (ever)	228 (76%)		

HAART use (ever)	209 (70%)		

HAART duration (y)*	5.0 (3.4–6.3)		

NRTI use (ever)	228 (76%)		

NRTI duration (y)*	6.9 (4.2–10.1)		

NRTI use (ever)	143 (48%)		

NNRTI duration (y)*	2.4 (1.0–4.0)		

PI use (ever)	195 (65%)		

PI duration (y)*	4.6 (3.0–6.1)		

Current CD4^+^ T cells/μL	434 (272–653)		

Nadir CD4^+^ T cells/μL	172 (50–329)		

Plasma HIV RNA (copies/mL)			

<75	159 (53%)		

75–1999	56 (19%)		

2000–9999	35 (12%)		

>10 000	50 (17%)		

Data are presented as median (IQR) or numbers (percent).

HAART, highly active antiretroviral therapy; IQR, interquartile range; CAD/CVA, cardiovascular/cerebrovascular disease; ARV, antiretroviral; NRTI, nucleoside reverse transcriptase inhibitor; NNRTI, nonnucleoside reverse transcriptase inhibitor; PI, protease inhibitor; TG, triglycerides; y, years.

* Duration of antiretroviral use among ever users.

† p<0.05.

**Table 3.** Baseline Characteristics of HIV-Infected and HIV–Uninfected Participants by Disease Category

Parameter	Elite Controller (*n*=26)	Treated Unsuppressed (*n*=71)	Treated Suppressed (*n*=135)	Untreated Unsuppressed (*n*=68)	HIV− (*n*=47)
Age (y)	50 (42–52)	45 (41–55)	49 (43–53)	44 (39–50)	43 (38–51)

Gender					

Male	20 (77%)	63 (89%)	127 (94%)	58 (85%)	38 (81%)

Female	6 (23%)	8 (11%)	7 (5%)	8 (12%)	9 (19%)

Transgender M->F	0	0	1 (1%)	2 (3%)	0

Race					

Caucasian	15 (58%)	39 (55%)	93 (69%)	34 (50%)	36 (77%)

African American	7 (27%)	20 (28%)	19 (14%)	29 (43%)	3 (6%)

Latino	3 (12%)	9 (13%)	14 (10%)	4 (6%)	4 (9%)

Other	1 (4%)	3 (4%)	9 (7%)	1 (1%)	4 (9%)

History of CAD/CVA	0	2 (3%)	14 (10%)	7 (10%)	1 (2%)

Cigarette smoking (ever)	21 (81%)	53 (75%)	84 (62%)	49 (72%)	24 (51%)

Diabetes mellitus	1 (4%)	7 (10%)	10 (7%)	3 (4%)	1 (2%)

Hypertension	8 (31%)	21 (30%)	38 (28%)	17 (25%)	4 (9%)

Hyperlipidemia treatment	4 (16%)	10 (14%)	36 (27%)	1 (1%)	1 (2%)

LDL (mmol/L)	2.46 (1.77–3.26)	2.64 (1.90–3.15)	2.69 (2.23–3.36)	2.51 (2.08–3.03)	2.80 (2.21–3.64)

HDL (mmol/L)	1.15 (0.90–1.31)	0.97 (0.77–1.18)	1.08 (0.85–1.31)	1.05 (0.90–1.23)	1.26 (1.08–1.56)

TG (mmol/L)	1.35 (0.97–1.91)	1.74 (1.10–2.80)	2.31 (1.42–4.24)	1.11 (0.78–1.45)	0.99 (0.73–1.59)

T Chol (mmol/L)	4.15 (3.51–5.56)	4.49 (3.67–5.36)	5.00 (4.46–5.90)	4.26 (3.62–4.85)	4.77 (4.15–5.72)

hsCRP (nmol/L)	14.29 (8.57–22.86)	13.33 (5.71–35.24)	19.05 (6.67–41.91)	20.00 (07.62–45.72)	10.48 (3.81–29.52)

BMI (kg/m^2^)	28 (26–33)	25 (23–28)	25 (22–28)	25 (23–29)	25 (23–30)

Hepatitis C virus	13 (50%)	18 (26%)	28 (21%)	16 (24%)	1 (2%)

antibody seropositivity					

Data are presented as median (IQR) or numbers (percent).

IQR, interquartile range; CAD/CVA, cardiovascular/cerebrovascular disease; TG, triglycerides.

### Association of HIV infection with Baseline Carotid IMT Levels

**Figure 1.**
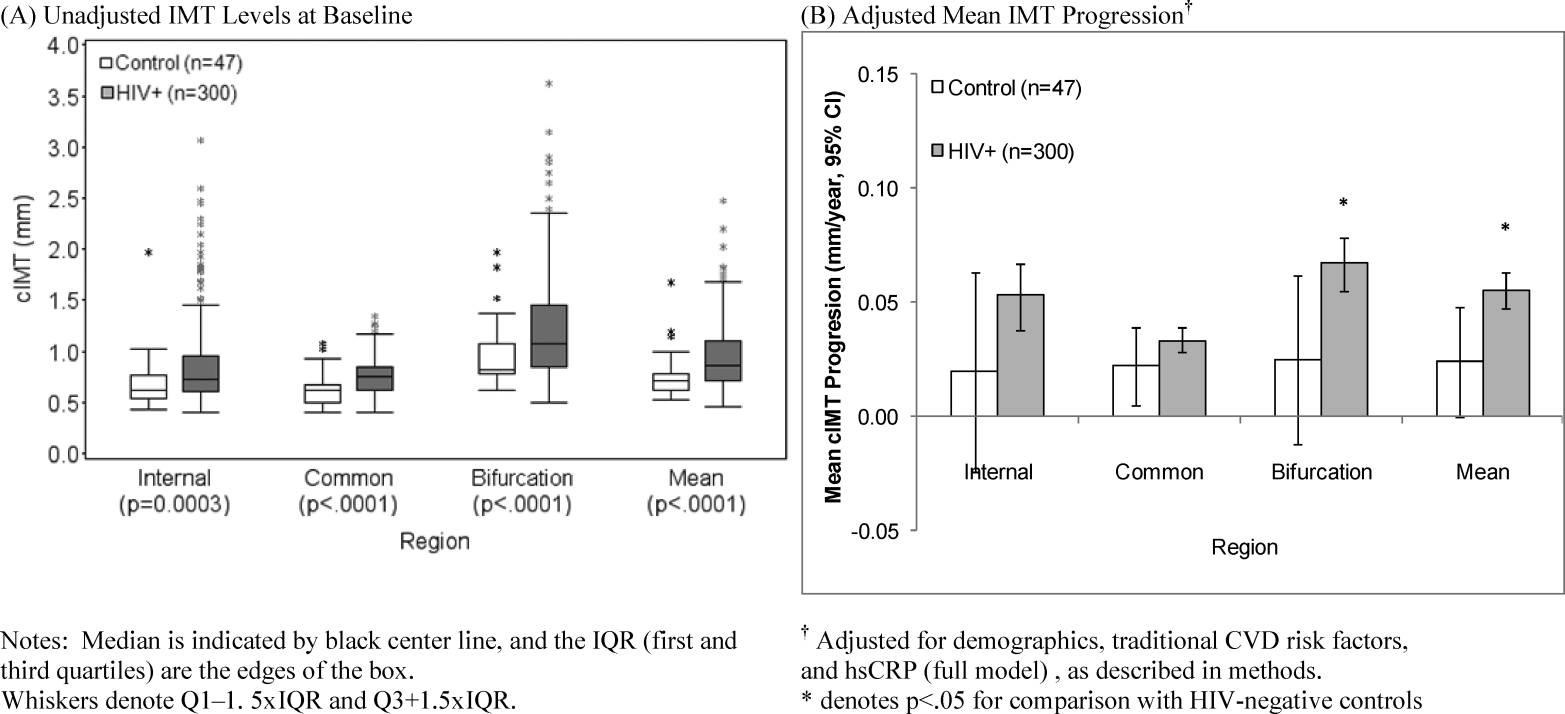
IMT Levels and Progression by Region and HIV status. (A) Unadjusted IMT levels at baseline: The IMT levels at baseline for all HIV-infected individuals were significantly higher as compared with uninfected controls at the internal, common, bifurcation region, and mean IMT (*P*<0.001 for all). (B) Mean IMT progression (adjusted): After adjustment for demographics, traditional factors, and hsCRP, IMT progression in the carotid bifurcation region and calculated mean IMT were higher among HIV-infected individuals as compared with controls, while there was no statistically significant difference in IMT progression in the internal and common carotid progression.

At baseline, mean levels of IMT were significantly higher in HIV-infected participants compared with controls at all sites (Figure 1A). After demographic adjustment (Table 4), HIV infection remained associated with higher overall IMT levels compared with control participants at all measured sites: internal (+0.176 mm, *P*=0.0037), common (+0.095 mm, *P*<0.0001), bifurcation (+0.257 mm, *P*=0.0003), and mean IMT (+0.158 mm, *P*=0.0002). This HIV association was unattenuated after adjusting for traditional CVD risk factors. Further adjustment for baseline hsCRP and change from baseline in hsCRP resulted in little change in the association of HIV infection with higher IMT (data not shown).

**Table 4.** Association of HIV-Infection with Overall Levels of Internal, Common, Bifurcation, and Mean IMT

IMT Measure	HIV-Infected (*N*=300†)	HIV− (*N*=47†)
Internal IMT (mm)		

Mean difference of HIV-infected vs. controls (95% CI)		

Adjusted for demographics‡	0.176 (0.058, 0.295)	

Adjusted for demographics and traditional CVD risk factors¶	0.175 (0.049, 0.302)	

Common IMT (mm)		

Mean difference of HIV-infected vs. controls (95% CI)		

Adjusted for demographics‡	0.095 (0.049, 0.141)	

Adjusted for demographics and traditional CVD risk factors¶	0.108 (0.059, 0.158)	

Bifurcation IMT (mm)		

Mean difference of HIV-infected vs. controls (95% CI)		

Adjusted for demographics‡	0.257 (0.117, 0.396)	

Adjusted for demographics and traditional CVD risk factors¶	0.251 (0.102, 0.400)	

Mean IMT (mm)		

Mean difference of HIV-infected vs. controls (95% CI)		

Adjusted for demographics‡	0.158 (0.075, 0.241)	

Adjusted for demographics and traditional CVD risk factors¶	0.158 (0.069, 0.247)	

Estimates are calculated from linear mixed models with random intercepts and slopes.

† *N* denotes number of participants included in analysis.

‡ Adjusted for age, sex, race, and time.

¶ Adjusted for age, sex, race, time, smoking status, diabetes, systolic blood pressure, diastolic blood pressure, total cholesterol, and HDL cholesterol.

### Rates of Change in IMT by HIV Infection Status

After adjusting for demographic, CVD risk factors, and hsCRP, HIV infection was associated with faster progression for mean IMT (+0.031 mm/year, *P*=0.016, Figure 1B and Table 5). This difference in IMT progression rates was most clearly seen in the bifurcation region (+0.040 mm/year, *P*=0.042). In contrast, the difference between HIV-infected and control participants in progression did not reach statistical significance for internal (+0.033 mm/year, *P*=0.16) and common IMT (+0.012 mm/year, *P*=0.20). Similar results were seen in more parsimonious models that retained only statistically significant factors (Table 6). Additional sensitivity analyses showed similar results when we excluded those with hyperlipidemia, diabetes, or hypertension.

**Table 5.** Multivariable Analysis of Factors Associated with Carotid Intima-Medial Thickness Progression in HIV-Infected and HIV− Uninfected Participants (Full Model)

Factor	Internal IMT (mm/year) Estimate (95% CI)	Common IMT (mm/year) Estimate (95% CI)	Bifurcation IMT (mm/year) Estimate (95% CI)	Mean IMT (mm/year) Estimate (95% CI)
Progression in HIV	0.053 (0.038, 0.067), *P*<0.0001†	0.033 (0.028, 0.039), *P*<0.0001†	0.067 (0.055, 0.078), *P*<0.0001†	0.055 (0.047, 0.063), *P*<0.0001†

Progression in HIV-controls	0.020 (−0.024, 0.063), *P*=0.37	0.022 (0.005, 0.039), *P*=0.012†	0.025 (−0.012, 0.062), *P*=0.19	0.024 (−0.000, 0.048), *P*=0.050

HIV vs. HIV-control (difference in rate of progression)*	0.033 (−0.013, 0.079), *P*=0.16	0.012 (−0.006, 0.031), *P*=0.20	0.040 (0.002, 0.078), *P*=0.042†	0.031 (0.006, 0.056), *P*=0.016†

Other factors in model:				

Age (per decade)	0.032 (0.016, 0.049), *P*=0.0002†	0.004 (−0.002, 0.011), *P*=0.21	0.012 (−0.001, 0.026), *P*=0.075	0.019 (0.010, 0.028), *P*<0.0001†

Female vs. male	0.025 (−0.025, 0.074), *P*=0.33	0.009 (−0.010, 0.029), *P*=0.35	0.004 (−0.038, 0.046), *P*=0.84	0.010 (−0.017, 0.036), *P*=0.47

African American vs. Caucasian	0.013 (−0.025, 0.051), *P*=0.50	0.008 (−0.008, 0.023), *P*=0.32	0.025 (−0.006, 0.056), *P*=0.11	0.014 (−0.006, 0.034), *P*=0.17

Latino/other vs. Caucasian	0.009 (−0.031, 0.049), *P*=0.66	0.007 (−0.009, 0.023), *P*=0.37	0.010 (−0.023, 0.042), *P*=0.56	0.007 (−0.014, 0.028), *P*=0.52

Diabetic	−0.033 (−0.091, 0.026), *P*=0.27	−0.011 (−0.033, 0.012), *P*=0.35	−0.068 (−0.113, −0.024), *P*=0.0030†	−0.039 (−0.069, −0.009), *P*=0.012†

Ever smoker	0.032 (0.003, 0.062), *P*=0.031	−0.001 (−0.013, 0.010), *P*=0.80	0.018 (−0.005, 0.041), *P*=0.13	0.016 (0.000, 0.031), *P*=0.044†

SBP (per 10 mm Hg)	−0.006 (−0.021, 0.009), *P*=0.45	0.002 (−0.004, 0.008), *P*=0.54	0.001 (−0.011, 0.013), *P*=0.83	−0.000 (−0.008, 0.008), *P*=0.97

DBP (per 10 mm Hg)	0.002 (−0.017, 0.022), *P*=0.83	0.003 (−0.005, 0.011), *P*=0.47	0.003 (−0.012, 0.019), *P*=0.68	0.003 (−0.008, 0.013), *P*=0.59

HDL (per 0.26 mmol/L)	−0.004 (−0.014, 0.007), *P*=0.50	0.000 (−0.004, 0.004), *P*=0.93	−0.002 (−0.011, 0.006), *P*=0.56	−0.001 (−0.007, 0.004), *P*=0.65

T Chol (per 0.26 mmol/L)	0.002 (−0.001, 0.005), *P*=0.13	0.001 (−0.001, 0.002), *P*=0.38	0.000 (−0.002, 0.003), *P*=0.85	0.001 (−0.001, 0.003), *P*=0.23

hsCRP (per doubling)	0.001 (−0.007, 0.008), *P*=0.87	−0.001 (−0.004, 0.002), *P*=0.61	0.005 (−0.001, 0.011), *P*=0.073	0.002 (−0.002, 0.006), *P*=0.30

Results reported as estimated IMT progression in mm/year (95% confidence interval).

Estimates are calculated from linear mixed models with random intercepts and slopes.

* HIV vs. HIV− uninfected control difference in progression is shown adjusted for traditional CVD risk factors and hsCRP.

† p < 0.05.

SBP, systolic blood pressure; DBP diastolic blood pressure.

**Table 6.** Multivariable Analysis of Factors Associated with Carotid Intima-Media Thickness Progression in HIV-Infected and HIV− Uninfected Participants (Parsimonious Models)

Factor	Internal IMT (mm/year) Estimate (95% CI)	Common IMT (mm/year) Estimate (95% CI)	Bifurcation IMT (mm/year) Estimate (95% CI)	Mean IMT (mm/year) Estimate (95% CI)
Progression in HIV	0.052 (0.039, 0.066), *P*<0.0001†	0.034 (0.029, 0.039), *P*<0.0001†	0.066 (0.055, 0.077), *P*<0.0001†	0.055 (0.048, 0.063), *P*<0.0001†

Progression in HIV-controls	0.017 (−0.022, 0.056), *P*=0.39	0.022 (0.006, 0.038), *P*=0.0072†	0.023 (−0.012, 0.057), *P*=0.19	0.021 (−0.001, 0.044), *P*=0.063

HIV vs. HIV-control (difference in rate of progression)*	0.035 (−0.007, 0.077), *P*=0.099	0.011 (−0.006, 0.028), *P*=0.21	0.045 (0.010, 0.080), *P*=0.012†	0.035 (0.012, 0.058), *P*=0.0034†

Other factors in model				

Age (per decade)	0.030 (0.015, 0.046), *P*=0.0002†			0.017 (0.009, 0.026), *P*=0.0001†

Diabetic			−0.062 (−0.105, −0.020), *P*=0.0047†	−0.033 (−0.062, −0.004), *P*=0.027†

Ever smoker	0.028 (0.001, 0.055), *P*=0.043†			0.017 (0.002, 0.032), *P*=0.025

hsCRP (per doubling)			0.007 (0.001, 0.013), *P*=0.016†	

Results reported as estimated IMT progression in mm/year (95% confidence interval).

Parsimonious models above remove statistically nonsignificant time × factor terms, but retain HIV status, time, demographics, traditional CVD, and hsCRP.

Estimates are calculated from linear mixed models with random intercepts and slopes.

* HIV vs. HIV− control difference in progression is shown adjusted for traditional CVD risk factors and hsCRP.

† p< 0.05.

Among HIV-infected individuals, the rate of progression in the bifurcation region was more rapid compared with the common carotid (+0.034 mm/year, 95% CI, 0.025–0.043, *P*<0.0001), internal (+0.014, 95% CI, 0.002–0.026, *P*=0.018), or mean IMT (+0.012, 95% CI, 0.003–0.021, *P*=0.0057). By contrast, rates of progression among the uninfected controls were similar at all sites, and tests of differences did not reach statistical significance (*P*>0.38 for all). As a sensitivity analysis, 5% of individuals with the largest outliers were removed, and the difference in rate of progression between HIV-infected and -uninfected individuals remained similar to the original analysis (as shown in Table 7).

**Table 7.** Effect of Removal of Outliers on Carotid Intima-Media Thickness Progression in HIV-Infected and HIV−Uninfected Participants

Factor	Internal IMT (mm/year) Estimate (95% CI)	Common IMT (mm/year) Estimate (95% CI)	Bifurcation IMT (mm/year) Estimate (95% CI)	Mean IMT (mm/year) Estimate (95% CI)
Original results				

Progression in HIV	0.053 (0.038, 0.067), *P*<0.0001†	0.033 (0.028, 0.039), *P*<0.0001†	0.067 (0.055, 0.078), *P*<0.0001	0.055 (0.047, 0.063), *P*<0.0001†

Progression in HIV-controls	0.020 (−0.024, 0.063), *P*=0.37	0.022 (0.005, 0.039), *P*=0.012†	0.025 (−0.012, 0.062), *P*=0.19	0.024 (−0.000, 0.048), *P*=0.050

HIV vs. HIV-control (difference in rate of progression)*	0.033 (−0.013, 0.079), *P*=0.16	0.012 (−0.006, 0.031), *P*=0.20	0.040 (0.002, 0.078), *P*=0.042	0.031 (0.006, 0.056), *PP* = 0.016

Results after removing outliers				

Progression in HIV	0.048 (0.037, 0.059), *P*<0.0001†	0.031 (0.026, 0.036), *P*<0.0001†	0.069 (0.059, 0.080), *P*<0.0001†	0.053 (0.047, 0.060), *P*<0.0001†

Progression in HIV-controls	0.017 (−0.016, 0.050), *P*=0.31	0.019 (0.005, 0.033), *P*=0.0092	0.029 (−0.002, 0.060), *P*=0.068	0.025 (0.004, 0.045), *P*=0.018

HIV vs. HIV-control (difference in rate of progression)*	0.031 (−0.005, 0.066), *P*=0.090	0.013 (−0.002, 0.028), *P*=0.099	0.037 (0.005, 0.070), *P*=0.026†	0.028 (0.007, 0.050), *P*=0.011†

* HIV vs. HIV–control difference in progression is shown adjusted for traditional CVD risk factors and hsCRP.

† p<0.05.

To address the question of whether a small group of individuals is driving the overall mean rate of progression, we looked at a number of influence statistics (including Cook's *D*, the PRESS statistic, and the likelihood distance) from the mixed linear regression model, accounting for the within-subject clustering. As a sensitivity analysis, we excluded participants with large influence statistics. When we removed the 5% of individuals with the largest outliers, the difference in rate of progression between HIV-infected and -uninfected participants remained similar to the original analysis, as shown above.

### Rates of Change in IMT by HIV Treatment Status and Disease Category

We also compared rates of IMT progression of HIV-infected participants by disease category. All four disease categories showed higher baseline IMT levels compared to controls (*P*<0.0001 vs. controls at all sites). All four disease categories appeared to have faster rates of overall IMT progression relative to controls, but after controlling for other risk factors, the differences reached statistical significance only for elite controllers (*P*<0.01 vs. controls for internal and mean) and treated unsuppressed patients (*P*<0.05 vs. controls for bifurcation and mean). The rates of progression within each region (common, internal, bifurcation) exhibited comparable trends to that observed for overall mean IMT. After adjustment for demographics, CVD risk factors, and hsCRP, rates of IMT progression were numerically higher in HIV-infected groups compared with the controls, although most differences were not statistically different. Similar results were seen in models that included disease category as a time-varying covariate.

### hsCRP and IMT Progression

**Figure 2.**
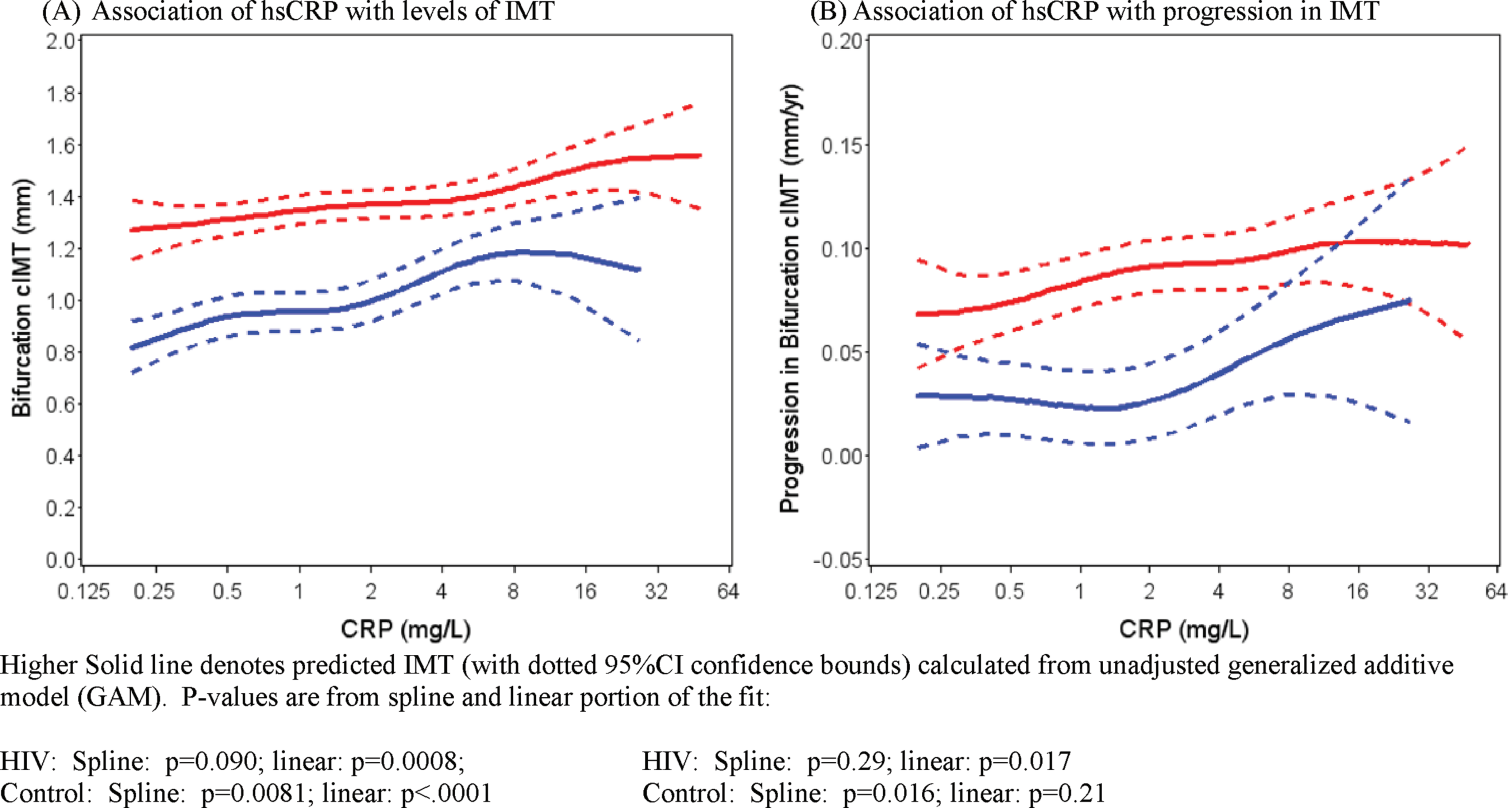
Unadjusted association of hsCRP with IMT levels and IMT progression in the bifurcation region: In the bifurcation region, higher hsCRP levels were associated with higher levels of IMT at baseline (A) and IMT progression over time (B) in both HIV-infected and HIV-uninfected participants. Higher solid line denotes predicted IMT (with dotted 95%CI confidence bounds) calculated from unadjusted generalized additive model (GAM). P-values are from spline and linear portion of the fit. A) HIV (red): Spline: p=0.090; linear: p=0.0008; Control (blue): Spline: p=0.0081; linear: p<.0001. B) HIV (red): Spline: p=0.29; linear: p=0.017 Control (blue): Spline: p=0.016; linear: p=0.21.

At baseline, hsCRP values were higher among all HIV-infected subjects (median 17.14 nmol/L, IQR, 7.62–41.91) compared with uninfected controls (median 10.48 nmol/L, IQR, 3.81–29.52, *P*=0.027). A graphical examination showed that increasing hsCRP appeared to be associated with both higher levels of IMT and faster progression in the bifurcation region (Figure 2). In a fully adjusted model (Table 5), there was a trend toward an independent association between hsCRP and bifurcation progression; this effect was stronger in the parsimonious model which demonstrated that hsCRP was independently associated with statistically significant progression in the bifurcation region (Table 6). The HIV effects were nearly the same in the full model as compared with the parsimonious model.

### Evaluation of plaque at baseline and followup

The prevalence of plaque at baseline in any segment was greater in HIV-infected individuals compared with uninfected participants (50% vs. 23%, *P*<0.0001); even after multivariable adjustment for traditional CVD risk factors and hsCRP, HIV infection remained associated with 2-fold higher risk of plaque (95% CI, 1.26–3.14, *P*=0.0033). HIV-infected subjects also had a higher prevalence of plaque compared with controls in the internal (23% vs. 6.4%, *P*=0.0037) and bifurcation (34% vs. 17%, *P*=0.014) segments, whereas the prevalence in the common segment was low in both groups (5.3% vs. 0%, *P*=0.14).

After excluding individuals with plaque present at baseline, HIV-infected individuals had a higher risk of incident plaque over the follow-up period (44% vs. 2.8%, *P*<0.0001). Similarly, the incidence of plaque was significantly higher in the internal (27% vs. 4.5% *P*=0.0007) and bifurcation segments 29% vs. 5.1%, *P*=0.0010) of HIV-infected persons compared with controls whereas little difference was seen in the common carotid region (8.1% vs. 2.1%, *P*=0.22).

## Discussion

CVD continues to be an important health concern among HIV-infected individuals.^27,28^ Although a number of studies have demonstrated that individuals with HIV infection are at increased risk for myocardial infarction,^2,29,30^ the underlying mechanisms associated with CVD and HIV infection remain to be described. Assessment of IMT using high-resolution ultrasound is a noninvasive technique that is predictive of future cardiovascular events and represents a direct quantification of subclinical vascular disease.^26^ Using this well-accepted measurement of atherosclerotic disease, we performed a comprehensive longitudinal assessment of IMT in a large heterogeneous cohort of HIV-infected and uninfected individuals, and found that HIV infection was independently associated with more rapid IMT progression in the carotid bifurcation region, with weaker associations in the other segments. Chronic inflammation—as defined by hsCRP levels at baseline—was elevated in HIV-infected subjects and was independently associated with IMT progression in the bifurcation region using a parsimonious model, although this effect was diminished in the fully adjusted model. HIV infection was independently associated with prevalence of plaque at baseline and was associated with greater incidence of plaque during follow-up; additionally, HIV-infected individuals had higher rates of plaque in the bifurcation and internal carotid regions as compared to controls. When considered in the context of emerging data from the general population, which suggest that the carotid bifurcation region is uniquely prone to the effects of chronic inflammation, our data are consistent with a model in which chronic HIV-associated inflammation causes accelerated atherosclerosis in the bifurcation region, and that this process collectively contributes to excess cardiovascular risk associated with HIV infection.

Carotid IMT studies in the HIV-infected population have yielded conflicting results. Initial reports showed that HIV-infected individuals on antiretroviral therapy and uninfected controls with similar metabolic disturbances had similar levels of IMT, as assessed by the right common carotid artery.^31^ In the ACTG 5078 study, no difference in IMT was detected among HIV-infected individuals and well-matched controls either at baseline^7^ or over time.^32^ All of these studies focused on assessment of the common carotid artery exclusively. In contrast, prior studies from our group (which included all segments) showed that HIV-infected individuals had higher IMT at baseline and at 1-year follow-up compared to controls.^5^ Both HIV infection and antiretroviral therapy were found to be associated with higher IMT in another large study;^4^ interestingly, the differences between HIV-infected and -uninfected groups were greatest in the carotid bifurcation region. Investigators from the FRAM study found that there was a stronger association of HIV infection with carotid IMT in the internal and bifurcation region than the common carotid.^6^ In the Women's Interagency HIV Study, when only the common carotid artery was assessed, no difference in mean IMT was found between HIV-infected and uninfected controls; however, frequency of carotid lesions, which included measurements of the internal and bifurcation region, was significantly increased among individuals with more advanced HIV disease.^8^ Our study extends these latter cross-sectional observations by showing differential progression of IMT over time. Individuals with HIV infection may be exposed to the effects of HIV for a much smaller proportion of their lifetime compared to chronic traditional risk factors such as cigarette smoking. As a result, in cross-sectional studies, the effects of HIV may be less prominent than other traditional risk factors, because the outcome measure is the absolute amount of IMT. This is in contrast to studies of IMT progression, which begin when the individuals is already infected with HIV and may allow more insight into the direct effects of HIV infection. Recently, in the SUN study, HIV-infected individuals with suppressed viral loads had decreased IMT progression assessed in the common carotid artery.^33^

The clinical implications of our observations remain to be defined. Early pathology studies have established that atherosclerotic lesions occur at vascular branch points.^34,35^ More specifically, among individuals without HIV infection, atherosclerotic lesions appear to occur at the carotid artery bifurcation.^9,36,37^ Vascular branch points are associated with low endothelial shear stress, which likely enhances atherogenesis through multiple mechanisms.^9–13,37–41^ Importantly, low endothelial shear stress may increase vascular susceptibility to the effects of chronic inflammation, perhaps by causing upregulation of adhesion molecules, chemokines, and pro-inflammatory cytokines.^16^ Our observation that changes in IMT are localized to areas known to be susceptible to the atherogenic effect of inflammation is broadly consistent with the observations that markers of inflammation are elevated in HIV disease and associated with cardiovascular events. ^18,42^ Among individuals without HIV infection in the Framingham Offspring Study, IMT of the internal carotid but not the common carotid significantly improved cardiovascular risk prediction, and authors of this study suggest that IMT of the internal carotid should be measured in addition to common carotid artery.^43^ Similar to our study, a single sonographer was used to perform all of the scans. Future studies will be needed to determine whether or not internal carotid IMT modifies CV risk in the setting of HIV infection.

We also explored the association of HIV-associated factors on IMT progression. To address this question, we have recruited a large number of individuals who have long-term HIV infection, but lack evidence of high-level viral replication and are not receiving potentially toxic antiretroviral drugs (elite controllers). As we previously reported, the elite controllers in this study had at baseline relatively high levels of overall IMT (indeed, the degree of IMT was comparable to that in the prototypic long-term treated HIV-infected subject).^21^ Our current study extends these finding and shows that elite controllers have faster rates of progression than HIV-uninfected controls, suggesting that HIV disease factors independent of treatment exposure, high-level viremia, and advanced immunodeficiency contribute to this process over time. We also found that optimally treated patients (ie, those with uninterrupted antiretroviral use maintaining an undetectable viral load) had no attenuation in the rate of progression in the bifurcation region. This finding suggests that fully effective therapy for HIV infection does not completely restore the rate of IMT progression to what is observed among uninfected controls.

Our study has several important limitations. Although this is a single-center study that allowed us to have great control over the imaging quality and imaging protocol, we recognize that accurate measurement of the carotid bifurcation region as well as internal carotid regions may be challenging for multicenter studies. An additional study limitation was that most of our covariates were measured only at baseline, which limited our ability to assess the effects of changes in risk factors and disease characteristics on IMT progression. An additional limitation is that our control group was much smaller than our HIV-infected group. However, our finding of greater IMT levels among HIV-infected individuals at baseline compared to controls is consistent with previous studies including the larger FRAM study^6^ and the study by Lorenz et al.^4^ Although some imaging protocols exclude plaques from IMT assessments, we measured IMT whether or not plaque was present which is the same method endorsed by the American Society of Echocardiography^26^, large observational cohorts,^44^ and clinical trials using IMT as the end point.^45^ For the purposes of our study, the distinction between plaque and IMT was not critical as we were evaluating IMT as a vascular marker of cardiovascular risk, and IMT in the presence or absence of plaque is an independent predictor of increased risk. Although there were differences in baseline characteristics between our HIV-infected and control participants, we found that levels of IMT and rates of progression remained increased in HIV-infected persons even after multivariable adjustment for demographic and traditional CVD risk factors. As is true with any observational study, unmeasured confounders may be present among our HIV-infected individuals, which distinguish them from uninfected controls, although we tried to adjust for all of the known risk factors.

## Summary/Conclusions

In conclusion, over a median follow-up period of 2.4 years, HIV infection and inflammation as assessed by hsCRP were both independently associated with more rapid IMT progression in the carotid bifurcation region, even after adjustment for traditional CVD risk factors. In contrast, there was no statistically significant difference in IMT progression in the internal and common carotid regions between HIV-infected individuals and controls. Development of plaque occurred more frequently in the bifurcation and internal carotid regions in HIV-infected individuals as compared with controls and was less frequent in the common carotid in both groups. As data from uninfected individuals demonstrate that the carotid bifurcation region is uniquely prone to inflammatory effects as a result of low endothelial shear stress, our findings suggest that the impact of HIV infection on vascular function may be most apparent in branch points due to the effects of inflammation. These observations may have important implications for future studies regarding the pathogenesis, epidemiology and management of HIV-associated CVD.
